# A High-Throughput Kinetic Assay for RNA-Cleaving Deoxyribozymes

**DOI:** 10.1371/journal.pone.0135984

**Published:** 2015-08-26

**Authors:** Jonas Eriksson, Henrik Helmfors, Ülo Langel

**Affiliations:** Department of Neurochemistry, Stockholm University, Stockholm, Sweden; CNR, ITALY

## Abstract

Determining kinetic constants is important in the field of RNA-cleaving deoxyribozymes (DNAzymes). Using todays conventional gel assays for DNAzyme assays is time-consuming and laborious. There have been previous attempts at producing new and improved assays; however these have drawbacks such as incompatibility with structured DNAzymes, enzyme or substrate modifications and increased cost. Here we present a new method for determining single-turnover kinetics of RNA-cleaving DNAzymes in real-time and in a high-throughput fashion. The assay is based on an intercalating fluorescent dye, PicoGreen, with high specificity for double-stranded DNA and heteroduplex DNA-RNA in this case formed between the DNAzyme and the target RNA. The fluorescence decreases as substrate is converted to product and is released from the enzyme. Using a Flexstation II multimode plate reader with built in liquid handling we could automate parts of the assay. This assay gives the possibility to determine single-turnover kinetics for up to 48 DNAzymes simultaneously. As the fluorescent probe is extrinsic there is no need for enzyme or substrate modifications, making this method less costly compared to other methods. The main novelty of this assay is the possibility of using full-length mRNA as the DNAzyme target.

## Introduction

RNA-cleaving DNAzymes are non-naturally occurring molecules first developed in 1994 by Breaker and Joyce through systematic evolution of ligands by exponential enrichment (SELEX) [1]. Further selection resulted in DNAzymes (‘8–17’ and ‘10–23’) capable of cleaving RNA under simulated physiological conditions [2]. DNAzymes generally comprise two binding arms reverse complementary to a target RNA and a catalytic core to induce cleavage of the target RNA, much like hairpin ribozymes, and are mainly composed of deoxyribonucleotides. The binding arms are usually between 7 and 17 nucleotides each [2,3], binding to the target RNA through Watson-Crick base pairing. Generally the kinetic constants of a DNAzyme are determined using short synthetic substrate that mimics the target region of the mRNA. Since mRNAs are large molecules with intramolecular bonds DNAzyme binding to the target site can be sterically hindered which will not be detected when using short mimic substrates. RNA-cleaving DNAzymes are generally dependent on divalent metal-ions for the cleavage of the substrate with varying affinity depending on the catalytic core of choice. This led to major research conducted in metal-ion detection and biosensing using DNAzymes [4–6]. Even intracellular detection using sodium-dependent DNAzymes has been developed [7]. DNAzymes have over the years been modified in many ways, mostly by inserting nucleotide analogs for increased stability and altered binding kinetics [8].

Ever since the first article on RNA-cleaving deoxyribozymes the enzyme kinetics have been determined mainly through a time-consuming method which is vulnerable to bias and pipetting errors as well as limited to low sample numbers [1]. To perform this conventional gel assay, reactions are set up and samples withdrawn and quenched, by metal chelators (i.e. EDTA), denaturing solvent (i.e. formamide) and freezing, at set time-points for further processing by gel electrophoresis and subsequent band quantification. Gel band quantification can be prone to user bias. A few attempts to find better methods have been made; *Kankia* published a method in 2006 where the substrate was modified to, after DNAzyme cleavage, produce a product forming a G-quadruplex which could be measured by UV [9]; In 2002 *Ferrari* and *Peracchi* published an article about a continuous kinetic assay for DNAzymes [10]. By using a fluorescence spectrometer and a stopped-flow apparatus they could measure the binding of ethidium bromide (EtBr), a fluorescent nucleic acid intercalator, to the enzyme-substrate complex forming DNA-RNA hybrid duplexes; following the fluorescence decrease, correlating to the concentration decrease of enzyme-substrate/product complex as product and EtBr gets released from the enzyme, over the time-course of the reaction. These methods constitute an evolution of the field but have one or several of the following drawbacks: can only measure single-turnover, substrate has to be modified, not high-throughput, highly structured DNAzymes are difficult to measure and full-length mRNA is not possible to use as substrate.

As full-length mRNA is not possible to use as a target for any of the above mentioned novel assays, choosing a target site for DNAzyme cleavage of an mRNA is not always trivial. mRNAs can be several thousands of nucleotides long, giving the possibility of tens to hundreds of potential DNAzyme target sites. To further add to the difficulty of DNAzyme design the binding arm length is something of a trial-and-error work to find the most efficient DNAzyme; a balance between binding affinity for the target and release from the product has to be achieved. Therefore one would have to test several lengths of binding arms increasing the number of possible DNAzymes, for targeting a single mRNA, to hundreds or thousands of different constructs. Structure prediction software could be an option for choosing a viable target site but they are not always accurate [11]. Another option is producing short RNA targets of each target site; however even without chemical or structural modifications it would be very costly. Some methods for target site choice have been published, such as mixing target mRNA with several DNAzymes with subsequent sequencing and quantification of cleavage at each site [12].

To address these drawbacks PicoGreen, an intercalating fluorescent dye with greater specificity for double-stranded oligonucleotides than EtBr, is introduced in the reaction vessel to monitor the concentration of DNAzyme:substrate complex by specifically binding to the formed double-stranded regions. The high fluorescence allows for a minimization of the reaction volume to allow for high-throughput screening together with a liquid-handling fluorescent plate reader (i.e. Flexstation II). Because of the high specificity of PicoGreen for double-stranded oligonucleotides and the low binding to RNA this new assay allows for determination of single-turnover kinetic constant of both structured DNAzymes as well as cleavage of full length mRNA substrates. With this method even the kinetics of a structured DNAzyme is measured efficiently and precisely in small volumes and low concentrations. In this article we solely use the ‘10–23’ DNAzyme [2].

## Materials and Methods

### Materials

All synthetic oligonucleotides were purchased from Eurofins. PCR primers were desalted, DNAzymes were purified by Eurofins proprietary High Purity Salt Free reverse phase cartridge purification system and short RNA substrates were purified by HPLC. RNase-free ultrapure water was produced using a Milli-Q Advantage A10 Water Purification System equipped with a BioPak® Polisher from Merck Millipore. Phusion High-Fidelity PCR kit and TranscriptAid T7 High Yield Transcription kit were from Life Technologies. 4-(2-Hydroxyethyl)piperazine-1-ethanesulfonic acid (HEPES), sodium chloride, manganese chloride tetrahydrate, formamide, bromophenol blue, 40% 19:1 acrylamide/bis-acrylamide, N,N,N′,N′-tetramethylethylenediamine, ammonium persulfate, urea, agarose, ethidium bromide and phenol:chloroform:isoamyl alcohol 25:24:1 pH 8.0 was purchased from Sigma-Aldrich. Ethylenediaminetetraacetic acid (EDTA) and sodium dodecyl sulfate (SDS) were from Scharlau. Boric acid was from VWR. Klorrent bleach was from Nilfisk. The nucleic acid dyes SYTO 61, PO-PRO 1, DRAQ5, Hoechst 33258, DAPI, Propidium iodide and PicoGreen were all from Life Technologies. GelRed was from Biotium. Ethidium bromide was from Sigma.

### Synthetic oligonucleotides

DNAzymes and short RNA substrates were resuspended in RNase-free ultrapure Milli-Q water and adjusted to 100 μM after measuring concentration using a NanoPhotometer P 300 (Implen), aliquoted and stored at -80°C. Working stocks were diluted to 10 μM and stored short-term at -20°C.

### In vitro transcription

PCR of pEGFP-C1 (Clontech) was performed using Phusion High-Fidelity PCR kit together with forward and reverse primers (Table 1) targeting eGFP regions 1–18 and 720–701, respectively, introducing a T7 promotor directly upstream of the eGFP gene. Full-length eGFP mRNA was obtained using the PCR-amplified template for *in vitro* transcription using TranscriptAid T7 High Yield Transcription Kit. DNA template was degraded by DNase I digestion and RNA transcript was purified by phenol:chloroform-extraction, ethanol-precipitated with a final concentration of 0.3 M sodium acetate and further resuspended in ultrapure Milli-Q water to 10 μM. RNA transcript length was confirmed by gel electrophoresis.

**Table 1:** Synthetic oligonucleotides.

	Sequence (5’-3’)	Ref
**DNAzymes**		
DzSJ	GCA CCC AGG CTA GCT ACA ACG ACT CTC TC	[2, 10]
I-DzSJ	GCA CCC AGG CTA **C**CT ACA ACG ACT CTC TC	
Dz451	GCC ATG ATA TAG AGG CTA GCT ACA ACG AGT TGT GGC TGT TG	
I-Dz451	GCC ATG ATA TAG AGG CTA **C**CT ACA ACG AGT TGT GGC TGT TG	
**RNA substrates**		
17 nt DzSJ substrate (start codon of HIV-1 gag-pol)	*GGA GAG AGA* | *UGG GUG CG*	[2, 10]
35 nt Dz451 substrate (eGFP nucleotides 434–468)	*ACU ACA ACA GCC ACA ACG* | *UCU AUA UCA UGG CCG AC*	
**PCR primers**		
Forward primer GFP (1–18) with T7 promotor	TAA TAC GAC TCA CTA TAG GG ATG GTG AGC AAG GGC GAG	
Reverse primer GFP (720–701)	TTA CTT GTA CAG CTC GTC CA	

DNAzymes, RNA substrates and PCR primers for reverse transcription. Underlined letters denote ‘10–23’ catalytic loop of DNAzymes; Bold letters are inactivating G-C mutations in catalytic loop; Italics are ribonucleotides; | represent the cleavage site.

### Conventional gel assay

To measure the observed kinetic constant of DNAzymes according to the conventional way the reactions were set up in 1.5 ml Eppendorf tubes as 100–150 μl reactions. Reactions contained 3 μM DNAzyme and substrate RNA, 50 mM HEPES (pH 7.4) and 75 mM NaCl. Reaction mixture was incubated at 25 or 37°C for 45 minutes before initiating the reaction by addition of 5 mM MnCl_2_ corresponding to 1/10^th^ of the final mixture volume. At different time-points 10 μl was withdrawn from the reaction mixture, mixed with 10 μl of stop solution (95% formamide, 18 mM EDTA, 0.025% SDS and 0.025% bromophenol blue) then stored at -20°C until all time-points had been collected. When using short RNA substrates the samples were run on 15 or 20% denaturing polyacrylamide gels containing 7 M Urea and 1X TBE while reaction samples containing full-length mRNA substrates were run on 3.5% TBE agarose gels containing 1% bleach [13]. Gels were post-stained using 1X GelRed and images were captured using a ChemiDoc XRS+ (Bio-Rad Laboratories). Images were analyzed and bands quantified using Image Lab (Bio-Rad Laboratories).

### Flexstation cleavage assay

Reaction mixtures were set up in 96 well half area black plates with clear bottom and NBS™-coating (Corning). Final concentration of DNAzyme and substrate in reaction mixtures was 1 μM each. All reactions were performed in 50 mM HEPES (pH 7.4), 75 mM NaCl. Final volume of each well was always 100μl. Each reaction mixture contained a final concentration of either 1 μM EtBr or 1:400 PicoGreen. Negative control wells were setup as the sample wells with the exception of either adding 10 mM EDTA or using an inactive version of the DNAzyme. Background wells were set up as the sample wells but excluding the substrate. Unused plate wells were kept sealed with plastic adhesive seal (MidSci) until use. Plates were preincubated at the temperature (i.e. 25 or 37°C) that the reaction was going to be carried out in for 45 minutes inside a Flexstation II multi-mode microplate reader. Reactions were initiated by addition of 10 μl (1/10^th^ of the final volume) 5 mM MnCl_2_ by the Flexstation fluidics module, directly after addition the sample wells were mixed through trituration. Measurements were taken every 30 seconds over the time-course of the experiments. EtBr experiments were excited at 300 nm and emission was captured at 605 nm using a cut-off filter at 495 nm. PicoGreen experiments were excited at 480 nm and emission was captured at 530 nm using a cut-off filter at 495 nm. Photomultiplier tube was set to ‘HIGH’ and number of reads per well was set to 30. The following dyes were also used in test experiments: GelRed, SYTO 61, PO-PRO 1, DRAQ5, Hoechst 33258, DAPI and Propidium iodide. Vapor-Lock PCR overlay was purchased from Qiagen. RiboLock RNase inhibitor was purchased from Life Technologies.

### Statistical analysis

All statistical analysis was made using GraphPad Prism (GraphPad Software). Background was subtracted from each data set which was then normalized to initial fluorescence (i.e. time point zero). Values are presented as means and error bars denote standard error of the mean.

## Results

**Fig 1:**
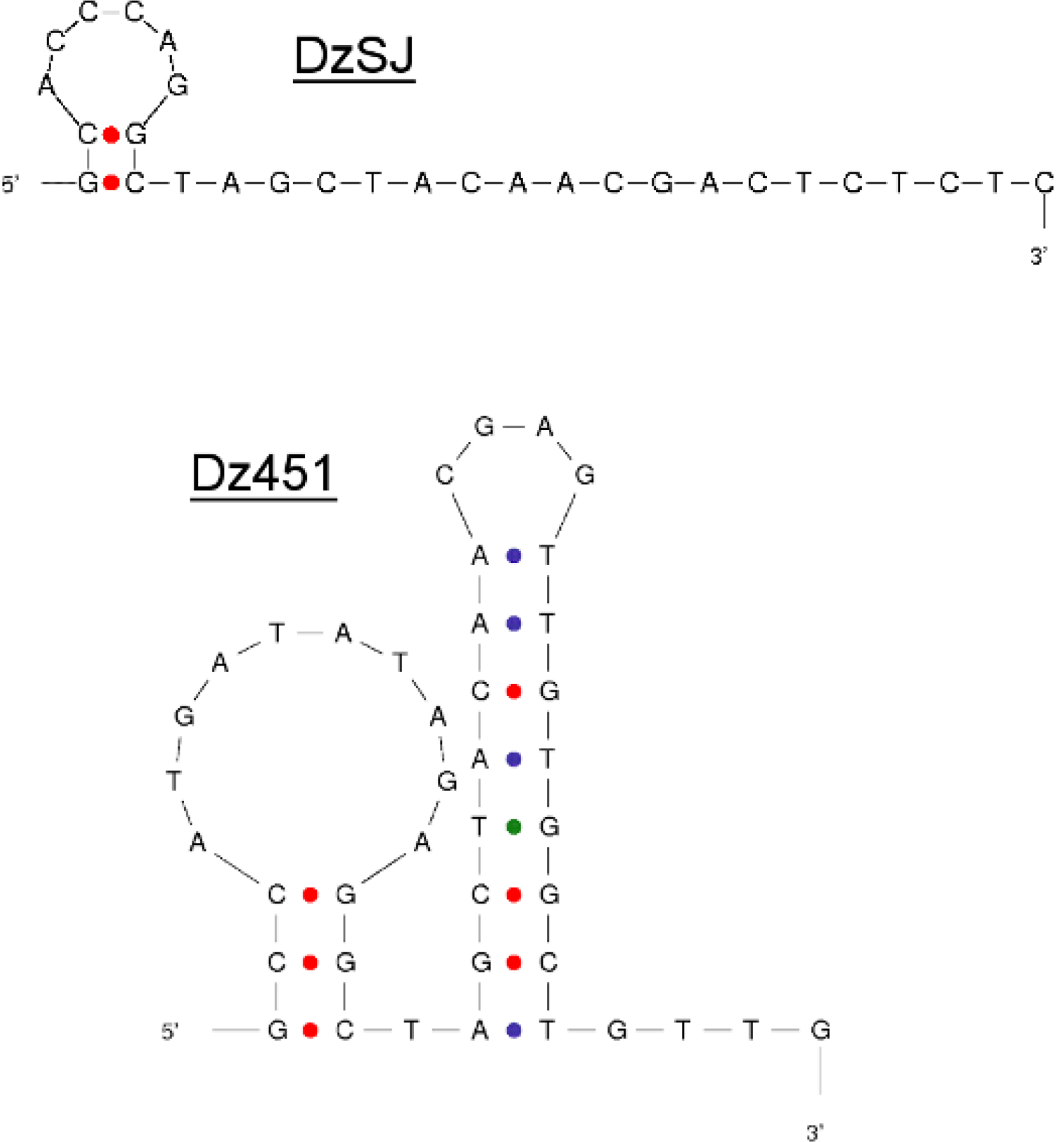
Predicted Mfold structures of DNAzymes. Predictions made at 25°C and 37°C for DzSJ and Dz451, respectively. Other parameters were set to 75 mM NaCl and 0.5 mM MgCl_2_. Shown here are the predicted structures with lowest ΔG. Intramolecular bonds are denoted by dots; red for G-C, blue for A-T and green for G-T.

Previous published method of using a fluorescent intercalator (EtBr) to measure DNAzyme kinetics [10] opened up many opportunities for improving and simplifying the kinetic measurements. However, there are several drawbacks in the method such as it not being optimized for high-throughput screenings and the inability to measure kinetics of highly structured DNAzymes, minimizing the number of possible DNAzymes applicable for this assay. We set a goal of developing a method that improves and address these drawbacks. We identified the main obstacles as signal variation and signal strength when using EtBr as a fluorescent intercalator together with a structured DNAzyme. Throughout this article we use two DNAzymes, one unstructured (DzSJ) and one structured (Dz451). In Fig 1 the Mfold predictions of each DNAzyme with lowest ΔG is shown [14]. Structure prediction was done for each DNAzyme at the respective assay temperature, i.e. 25 and 37°C, and salt concentrations set to 75 mM NaCl and 0.5 mM MgCl_2_.

**Fig 2:**
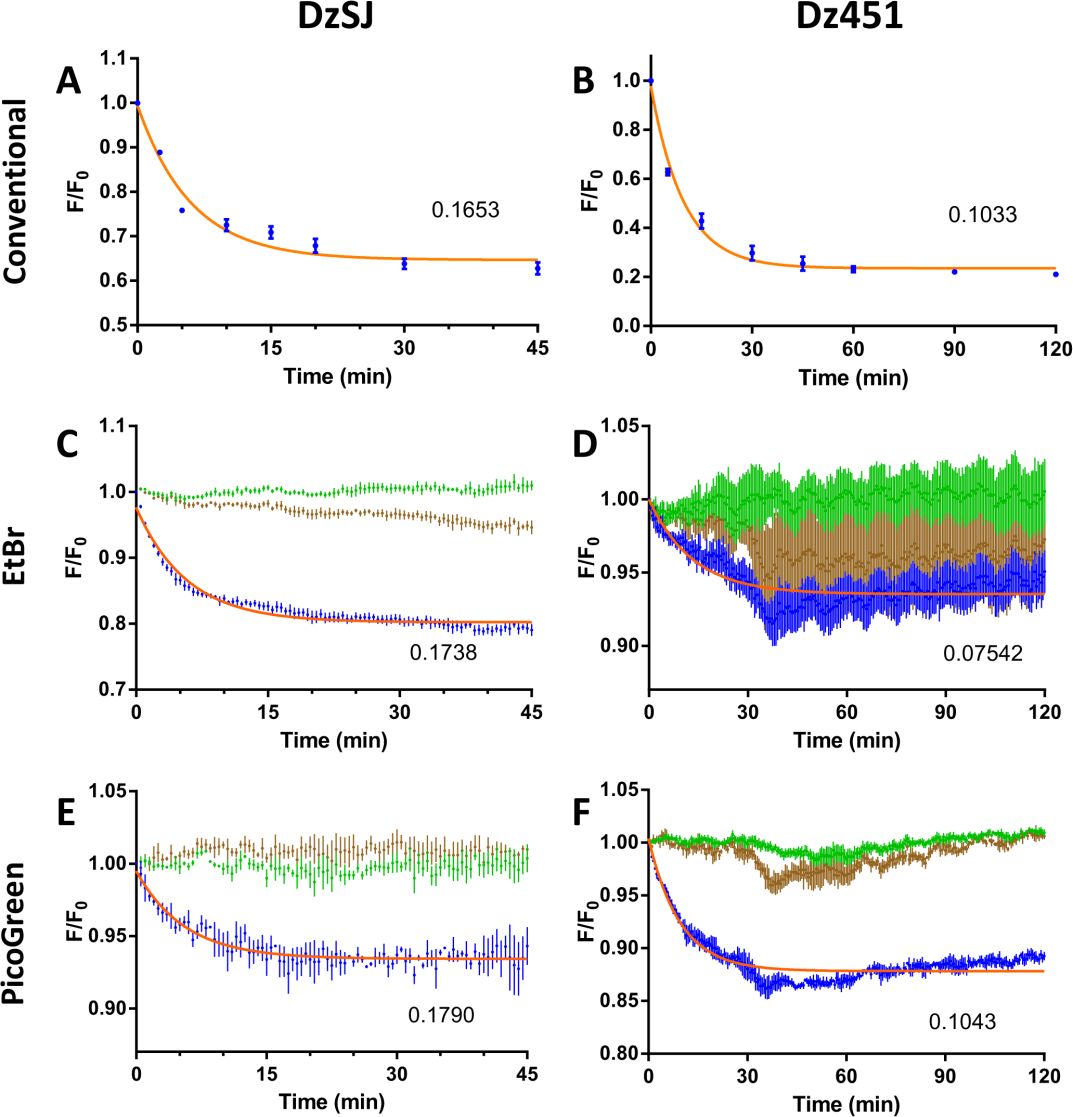
Comparison of three methods for single-turnover kinetics of two DNAzymes, DzSJ and Dz451. Kinetic analysis of DzSJ (**A**, **C** and **E**) and Dz451 (**B**, **D** and **F**) by three different methods: conventional gel assay (**A** and **B**), EtBr plate assay (**C** and **E**) and PicoGreen plate assay (**D** and **F**). The fitted k_obs_-value for decay curves are shown as min^-1^ inserted in each respective graph. Blue dots indicate DNAzyme with substrate, green dots control wells with EDTA and brown dots inactive DNAzyme controls. Orange line represents one phase decay fitting. Data shown as fluorescence mean normalized to initial data-point and control wells without RNA target ± SEM of three independent experiments, each performed in triplicate.

Control reactions were set up as the DNAzyme cleavage reaction additionally containing 10 mM EDTA to bind any divalent metal-ions thus preventing the enzymatic reaction. Also, inactive versions of each DNAzyme were included as an additional control. Inactivation was attained through a G-to-C substitution in the 6^th^ position of the catalytic loop [15]. Determining the single-turnover constants by conventional gel assay for each DNAzyme with a short synthetic RNA substrate which mimics the target site found on each respective full-length mRNA a k_obs_-value very similar to the published ones was acquired for DzSJ and a similar value for Dz451 (Fig 2A and 2B). Single-turnover kinetics were also determined using EtBr as an extrinsic probe and a Flexstation II multimode reader equipped with an 8-channel fluidics module to initiate the reaction by introducing 5 mM MnCl_2_ as 1/10^th^ of the final reaction volume (final conc. 0.5 mM). Reacting DzSJ and Dz451 with corresponding short target RNAs a very similar k_obs_, for the unstructured DzSJ, to the already acquired value from the conventional method was found (Fig 2C) while Dz451 produced an inaccurate k_obs_-value with high variance (Fig 2D).

To circumvent the issue several fluorescent dyes (GelRed, SYTO 61, PO-PRO 1, DRAQ5, Hoechst 33258, DAPI, Propidium iodide and PicoGreen) were screened and only one dye, PicoGreen, which gave both higher signal strength and lower signal variance than EtBr without inhibiting the DNAzyme cleavage reaction was found (Fig 2C–2F). PicoGreen is a double-stranded DNA-specific intercalating dye which exhibits a >1000-fold fluorescence increase when in complex with dsDNA or DNA-RNA heteroduplex [16]. PicoGreen has, unlike EtBr, very low affinity for single-stranded oligonucleotides, RNA and proteins.

**Fig 3:**
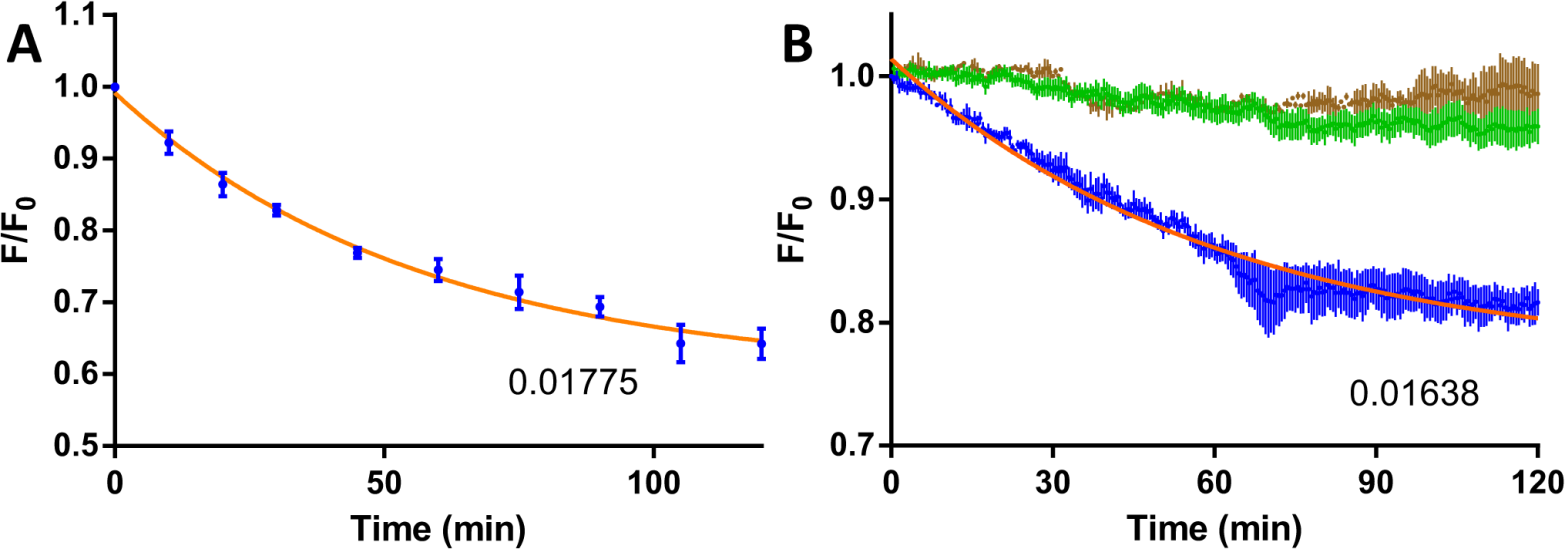
Comparison of conventional gel assay and PicoGreen plate assay of Dz451 cleaving full-length eGFP mRNA. Conventional gel assay (**A**) and PicoGreen plate assay (**B**). The fitted k_obs_-value for decay curves are shown as min^-1^ inserted in each respective graph. Blue dots indicate DNAzyme with substrate, green dots control wells with EDTA and brown dots inactive DNAzyme controls. Orange line represents one phase decay fitting. Data shown as fluorescence mean normalized to initial data-point and control wells without RNA target ± SEM of three independent experiments, each performed in triplicate.

Substituting a full-length mRNA with short RNA substrates is however not feasible since unlike the short synthetic variant, full-length mRNA has many intramolecular interactions which might alter the DNAzyme kinetics. Screening of DNAzymes targeting different regions of an mRNA is neither feasible when performing the conventional gel assay (not high-throughput) nor when using EtBr as an extrinsic probe (incompatible with structured DNAzymes). Fig 3A shows the conventional assay of Dz451 cleavage of full-length eGFP mRNA with a k_obs_-value of 0.01775 min^-1^ similar to previously published DNAzymes targeting full-length mRNAs [17] and about a fifth of the value acquired when using short RNA substrate. Using the same setup as for the DNAzyme reactions with short synthetic RNA substrate we confirmed the applicability of the assay for full-length mRNA kinetics even when using structured DNAzymes (Fig 3B). The k_obs_-values obtained through these assays have a difference of less than 8%.

In Table 2A comparison between k_obs_-values of each DNAzymes with respective targets and each assay setup is depicted.

**Table 2:** Comparison of k_obs_-values of each DNAzyme with respective target RNA for each kinetic assay.

	Gel assay /min^-1^	R^2^	EtBr assay /min^-1^	R^2^	PicoGreen assay /min^-1^	R^2^
DzSJ with short substrate	0.1653 ± 0.0231	0.96	0.1738 ± 0.0072	0.91	0.1790 ± 0.0214	0.60
Dz451 with short substrate	0.1033 ± 0.0092	0.98	0.07542 ± 0.00892	0.30	0.1043 ± 0.0041	0.79
Dz451 with full-length mRNA	0.01775 ± 0.00306	0.95	n.d.	n.d.	0.01638 ± 0.00094	0.89

Data is presented as k_obs_ ± standard error of the mean. n.d. = not determined.

## Discussion

A method for determining single-turnover kinetics of RNA-cleaving DNAzymes in real-time has been devised and presented in this article. The method is high-throughput and has addressed the drawbacks of previously published methods [9,10]. PicoGreen was shown to have much less signal variance than EtBr, thus even the kinetics of structured DNAzymes where the fluorescence difference of enzyme-substrate complex and free enzyme and product is low can be determined. This opens up the possibility to perform mass-screening of DNAzymes targeting the same mRNA to find the most efficient one in a high-throughput fashion.

The major drawback of this method is the incompatibility with multiple turnover kinetics. Since the method measures the enzyme-substrate complex concentration, a multiple turnover setup would have a constant signal, as this concentration does not change. The PicoGreen assay should be applicable with other kinds of RNA-cleaving DNAzymes. However, ribozymes are most likely not suitable for this method as PicoGreen has a very low affinity for RNA, even in its double-stranded form [16].

When comparing the results we obtained by using two different dyes we saw an approximate 10 times higher signal for PicoGreen compared to EtBr. The signal variance was significantly lower for the structured DNAzyme when using PicoGreen, which could be attributed to the >100 times lower K_d_ of PicoGreen to double-stranded DNA compared to EtBr [18]. The kinetic constants from each of the three methods used demonstrate that EtBr is a poor choice of dye for a high-throughput method as the kinetics could not be determined accurately for a structured DNAzyme (Table 2). The possibility of screening different DNAzymes targeting full-length mRNA is achieved here; with the addition of PicoGreen even the small change in fluorescence in the reaction of Dz451 and full-length eGFP mRNA can be measured with low variance. The labor-intensiveness of the conventional gel assay is removed in this assay owing to automatic pipetting and real-time continuous monitoring of reactions.

During the development of the assay several questions and problems were addressed. To minimize unspecific binding of oligonucleotides and PicoGreen to the plate Nonbinding surface (NBS™) coated plates were used. Since the plates are not covered by lids or films there is an increased risk of RNase-contamination; however no measurable RNase-contamination could be detected as negative controls had low or no decrease in fluorescence. Addition of RiboLock RNase inhibitor to 1 u/μl was tested without interfering with the assay (data not shown). EDTA-treated control wells proved the dependence of M^2+^ for the enzymatic cleavage to occur while the inactive DNAzyme controls show the reaction is not caused by contaminating nucleases and that the ‘10–23’ catalytic loop is essential. Another problem that could arise with an open system like this is evaporation. We did not see any major evaporation over 2 hours. However we did test Vapor-Lock, a hydrophobic PCR overlay, as an overlay to prevent evaporation when performing longer test runs. Vapor-Lock completely prevented evaporation and inferred no change of the fluorescence readout (data not shown). The fluorescent signal strength of intercalating dyes such as EtBr and PG are influenced by temperature. At 37°C we could see up to a 30% fluorescence drop over the first 30 minutes when initiating the reaction outside of the Flexstation II since the plate and its contents increased from room temperature to 37°C. This is an important observation and it stresses the importance of using a plate reader with integrated fluidics. Therefore we chose to use the Flexstation II which contains a fluidics module with an 8-channel pipettor for 96-well plates. Flexstation II can easily be modified to pipet and measure 384-well plates while the method presented here most likely also works in a smaller volume setup such as 384-well plates.

The method presented here opens up for high-throughput screening and continuous single-turnover kinetics determination of DNAzymes in real-time owing to the use of a fluorescent plate reader with integrated fluidics (i.e. Flexstation II) which previously has not been presented. The possibility to screen for the best candidate DNAzymes of targeting an mRNA decreases the labor-intensiveness compared to the conventional gel assay and also open up the possibility for a screening of all the possible DNAzymes targeting a single mRNA. With the ability of determining kinetic constants for unstructured and structured DNAzymes, the low variance and good reproducibility, this method is a superior alternative to the conventional gel assay and other methods previously published.
